# Safety and efficacy of fecal microbiota transplantation (FMT) as a modern adjuvant therapy in various diseases and disorders: a comprehensive literature review

**DOI:** 10.3389/fimmu.2024.1439176

**Published:** 2024-09-26

**Authors:** Mehdi Karimi, Niyousha Shirsalimi, Zahra Hashempour, Hossein Salehi Omran, Eshagh Sedighi, Farzan Beigi, Masoud Mortezazadeh

**Affiliations:** ^1^ Bogomolets National Medical University (NMU), Kyiv, Ukraine; ^2^ Faculty of Medicine, Hamadan University of Medical Science (UMSHA), Hamadan, Iran; ^3^ School of Medicine, Shiraz University of Medical Sciences (SUMS), Shiraz, Iran; ^4^ School of Medicine, Shahid Beheshti University of Medical Sciences (SBMUS), Tehran, Iran; ^5^ Department of Veterinary Medicine, Islamic Azad University Branch of Urmia, Urmia, Iran; ^6^ Students Research Committee, Arak University of Medical Sciences, Arak, Iran; ^7^ Department of Internal Medicine, Sina Hospital, Tehran University of Medical Sciences (TUMS), Tehran, Iran

**Keywords:** fecal microbiota transplantation, fecal transplantation, microbiota, dysbiosis, gut microbiome, immunomodulation, adjuvant therapy

## Abstract

The human gastrointestinal (GI) tract microbiome is a complex and all-encompassing ecological system of trillions of microorganisms. It plays a vital role in digestion, disease prevention, and overall health. When this delicate balance is disrupted, it can lead to various health issues. Fecal microbiota transplantation (FMT) is an emerging therapeutic intervention used as an adjuvant therapy for many diseases, particularly those with dysbiosis as their underlying cause. Its goal is to restore this balance by transferring fecal material from healthy donors to the recipients. FMT has an impressive reported cure rate between 80% and 90% and has become a favored treatment for many diseases. While FMT may have generally mild to moderate transient adverse effects, rare severe complications underscore the importance of rigorous donor screening and standardized administration. FMT has enormous potential as a practical therapeutic approach; however, additional research is required to further determine its potential for clinical utilization, as well as its safety and efficiency in different patient populations. This comprehensive literature review offers increased confidence in the safety and effectiveness of FMT for several diseases affecting the intestines and other systems, including diabetes, obesity, inflammatory and autoimmune illness, and other conditions.

## 1 Introduction

Recently, research has focused on the human gut microbiome to understand better its role and the need to control it for medical purposes. The human digestive system consists of trillions of microorganisms, collectively called the gut microbiome, playing various roles, such as digesting food, preventing diseases, and maintaining general health (1). Disruption in the gut microbial community is called dysbiosis, which can result in different health issues (2). Fecal microbiota transplant (FMT) is a therapeutic intervention that involves transferring fecal material from a healthy donor to a recipient with the primary aim of restoring the gut microbiota’s balance (3). FMT has become popular in recent years with the prospect of curing various conditions with high cure rates (about 80%-90%) (4).

Animal studies on FMT have explored its potential as a therapy and its impact on the gut microbiota, host immune response, and disease outcomes, serving as a preclinical model for human trials (5, 6). The therapeutic potential of FMT is a subject of ongoing studies, which will lead to further progress in this field.

Despite evidence of its therapeutic benefits and impact on our understanding of the microbiome, FMT faces numerous regulatory and safety challenges. Additional investigations and clinical trials can help establish FMT as a widely accepted therapeutic option for enhancing the lives of individuals with different diseases. This study aims to comprehensively review the current literature on FMT as a modern procedure for treating various diseases. We delve into investigations on the safety and efficacy of FMT in different disease entities, from intestinal disorders to non-intestinal ones, such as diabetes, hepatitis, obesity, and immune-mediated disorders.

## 2 History of FMT

The origins of FMT trace back to ancient China in the 4th century, when human fecal material, referred to as “yellow soup,” was utilized to address severe diarrhea in patients (7). The first recorded instance in modern medicine emerged in the 1950s, documented by Eiseman and colleagues, who effectively treated patients with pseudomembranous colitis using FMT (8). The contemporary understanding of FMT developed in the 20th century but remained relatively obscure for decades. Its popularity resurged in the 21st century as scientists recognized its potential in treating gastrointestinal disorders, especially recurrent clostridium difficile infection (CDI). In a 2013 study, Dr. Van Nood and collaborators reported a remarkable success rate of over 90% for recurrent CDI treated with FMT (9). Subsequent case studies have shown high success rates of using FMT in treating various diseases, including non-infectious diseases. The earliest documented application dates back to a 1989 study where a 45-year-old male with treatment-resistant ulcerative colitis experienced complete and sustained clinical recovery (10). Although FMT still faces regulatory and safety challenges, its historical trajectory highlights a significant shift in the current perception of the human microbiome and potential microbial therapies.

## 3 Preparation and procedure of FMT

**Figure 1:**
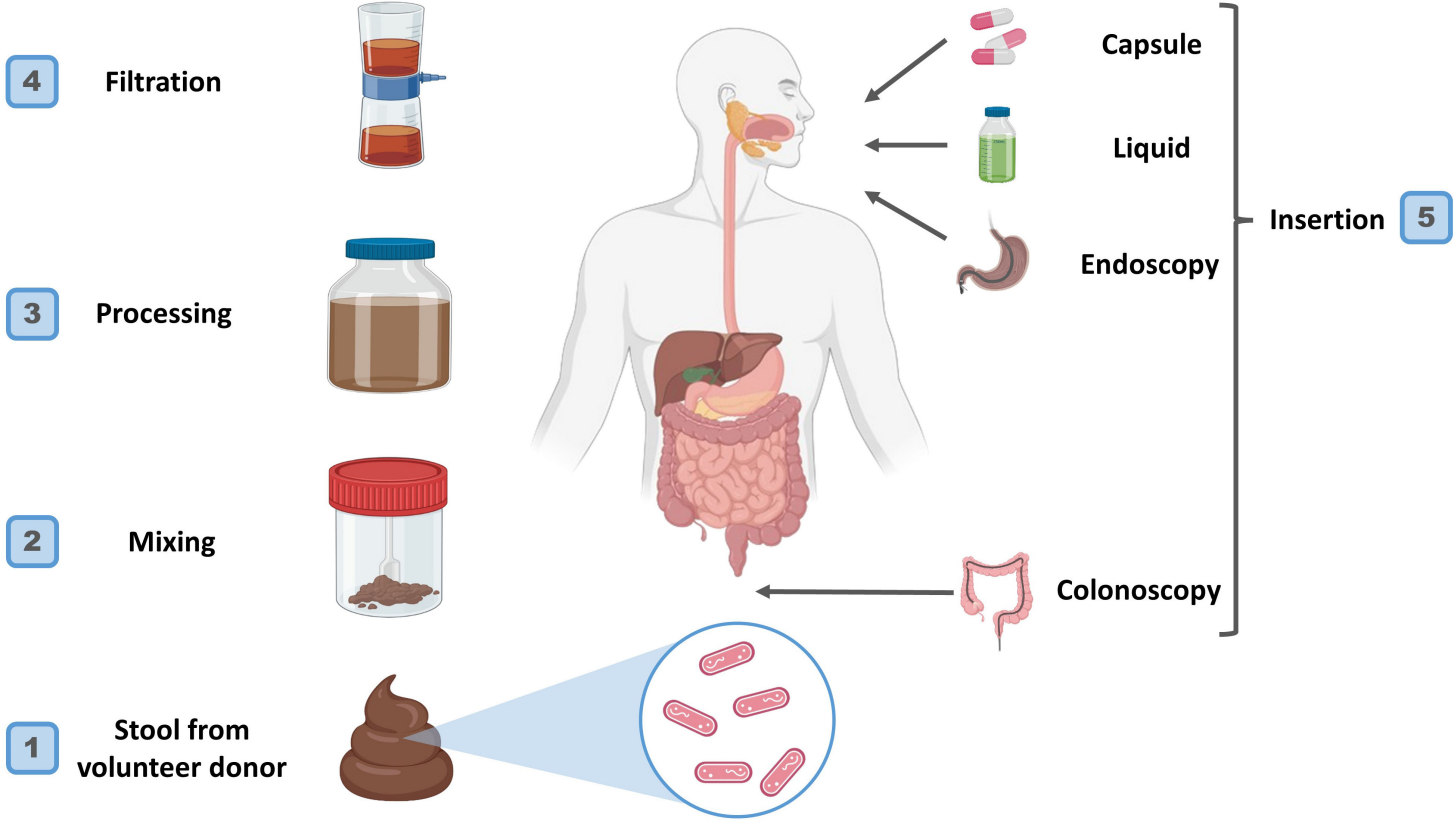
Preparation and procedure and rout of administration of FMT.

The main steps in preparing and applying FMT are donor selection, collection and processing of fecal material, freezing, storage, administration route selection, recipient preparation, and transplantation (11–14). It is essential to store microbiomes under proper conditions for their viability and composition. The donor’s fecal sample is collected and processed through various stages, filtered or diluted to form a standardized suspension. Some studies have investigated ways to stabilize fecal materials, such as using microcrystalline cellulose particles or lyophilization (freeze-drying) that enables storage or administration (11, 12). Processed feces may be cryopreserved to increase the availability of FMT while solving possible technical issues (14) (see **Figure 1**).

FMT can be administered through colonoscopy, nasogastric/nasoduodenal tube, or enema; choosing the route depends on the patient’s condition as well as personal preferences (15).

## 4 Mechanisms of action of FMT

FMT works via several key essential mechanisms, starting with the transfer of a diverse population of beneficial microbes from a healthy donor into the recipient’s GI tract (16). Initially, this new microbiota competes with and displaces harmful bacteria (dysbiosis), bringing the gut environment back into healthy gut microbiome balance (17). The process begins by suppressing the growth of dangerous bacteria such as *Clostridioides difficile*, which is a frequent target of FMT. The introduction of donor microbiota aids in the reestablishment of healthy levels of short-chain fatty acids (SCFAs), such as butyrate, which are essential for gut barrier integrity and immune system modulation. This microbial balance restoration is critical for reducing inflammation and improving gut health (16–19).

In the next stages, the donor microbiota is colonized and stabilized in the recipient’s gut over time. Over time, the transplanted microbiota combines with the host’s native bacteria, resulting in a more resilient and diversified microbial community. This diversity is essential for the gut’s normal function, including digestion, nutrition absorption, and immunological control. Furthermore, the newly established microbiota can generate bioactive substances that promote gut health and protect against future infections. This continual interaction between the transplanted and indigenous microbiota ensures a long-term therapeutic effect, helping to resolve symptoms and reduce the occurrence of disorders such as recurrent C. difficile infection (16–19).

Despite this procedure’s effectiveness, the exact mechanisms of action are not yet fully understood. It seems that FMT works by changing diversity and establishing microbiota, modulating the immune system (16, 20, 21). Several studies demonstrated that FMT restores microbial diversity, changes in metabolic functions, modulates the immune system (20–22), affects bacteriophage populations in the gut (23), influences the dynamics of bacterial strains (24), and may even impact neurological (25) and vascular diseases (26). A combination of these factors contributes to the mechanisms behind FMT’s efficacy.

FMT introduces a wide range of microorganisms that can re-establish a healthy microbial community (20, 22). It can also be efficient by impacting the abundance and persistence of specific bacterial strains within the gut (24). However, the use of broad-spectrum antibiotics alongside FMT, except those used for transplant preparation, can lead to its failure (21). Certain gut microbiota components induce the production of immune-modulatory compounds that help regulate the immune response. Therefore, FMT can also influence the immune system. It becomes particularly important in conditions characterized by inflammation, such as inflammatory bowel disease (IBD) (21, 22). Another instance is that during active, refractory graft-versus-host disease of the gastrointestinal tract, T-cell infiltration increases, which FMT can reduce (21). Additionally, studies suggest that FMT affects the occurrence and development of cerebrovascular diseases through systemic inflammatory immune responses (26).

**Figure 2:**
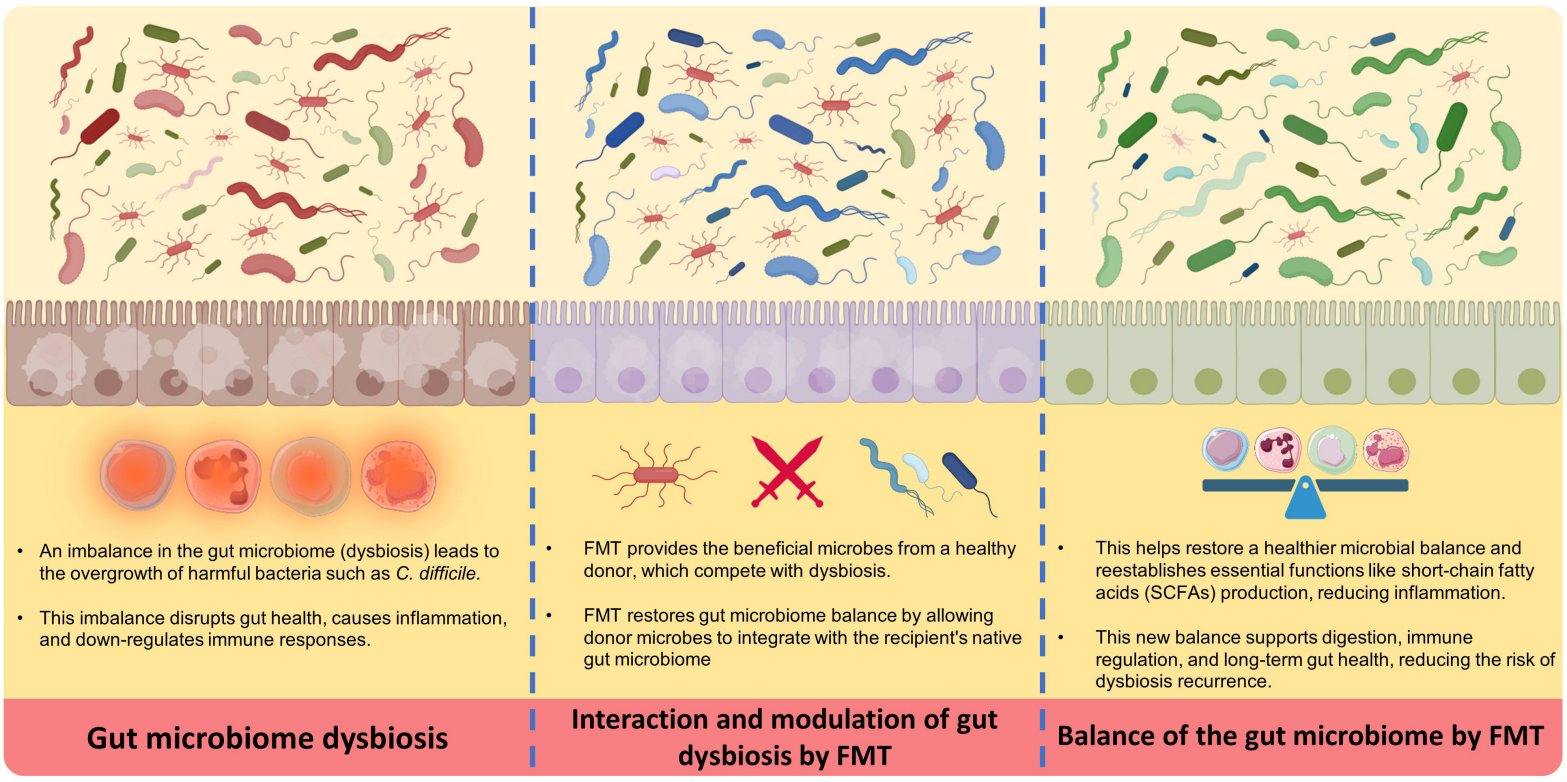
Mechanisms of action of FMT.

**Figure 2** Demonstrates a summary of the mechanism of action of FMT.

## 5 Importance of donor selection for FMT efficacy

Potential donors must be thoroughly evaluated to ensure the procedure’s safety and effectiveness. This includes screening for infectious diseases, antibiotic use, and other factors affecting the gut microbiome (11, 12). In some cases, the recipient may undergo a bowel preparation or receive antibiotics before FMT to help clear the existing microbiome and improve the chances of successful engraftment of the donor microbiome (11–14).. Evidence suggests that careful donor selection, standardized processing, and appropriate delivery can help ensure the safety and efficacy of FMT preparation and application (13, 27).

The guidelines for selecting and evaluating “stool donors” were initially based on blood donor guidelines, although certain tested pathogens in blood donation are typically not transferable through stool (28). The microorganisms transmitted during FMT can contain harmful pathogens. Intestinal dysbiosis is linked to an increasing number of diseases, including infectious, metabolic, cardiovascular, autoimmune, and neurologic conditions (29). Safety standards indicate that individuals with such pathologies must be excluded as stool donors to prevent the transfer of dysbiotic microbiota. Screening and excluding donors based on the presence of these diseases is essential to ensure the safety and efficacy of FMT (30, 31). These factors aim to enhance transplant success and minimize complications. Despite different variations between clinical settings and research studies, donor selection guidelines are necessary to mitigate risks and maximize FMT’s benefits for recipients (30, 31).

Donor selection for FMT is a complex process that involves detailed questionnaires, medical tests, and screening for infectious diseases to ensure safety and efficacy (32). The selection of a donor depends on various factors, including age, body mass index (BMI), genetic factors, general health, lifestyle and dietary habits, microbiome composition, and screening for chronic conditions (32–34). Despite the lack of specific guidelines for donors’ “age” criteria, a minimum age of 18 is recommended, as gut microbial diversity stabilizes by this age (34). Younger donors, ideally under 50 years old, are preferred (32), and obese donors (BMI over 30) or those with moderate to severe malnutrition are disqualified based on the BMI criteria (35, 36). Genetic factors shape the intestinal microbiota and metabolic phenotypes, as the microbiota of homozygotic twins are more comparable than those of dizygotic twins (37).

The food we eat has a big impact on the makeup and activity of the microorganisms in our gut, and it’s crucial for how our bodies interact with these microorganisms (38). Research shows that around 20% of changes in the structure of these microorganisms can be linked to diet, highlighting the possibility of using dietary changes to help manage diseases (39).

## 6 Indication and contraindications

Some case reports have shown the effectiveness and safety of FMT in conditions previously considered contraindications, such as sepsis, active massive gastrointestinal bleeding, perforation, severe intestinal damage, fulminant colitis or toxic megacolon, severe diarrhea, significant intestinal narrowing, high-output intestinal fistula, intolerance to enteral nutrition, immunodeficiency, recent use of high-risk immunosuppressants, and pregnancy or lactation (40–42).

In 2015, Li and colleagues published the first report on the use of FMT in treating a case of persistent sepsis and watery diarrhea after vagotomy. The patient experienced complete resolution of symptoms following FMT (43). Also, Wei et al. reported successful treatment with FMT in two septic shock patients following cerebrovascular stroke (41). In another report of three patients with ongoing symptoms of systemic inflammatory response (SIRS) and diarrhea leading to sepsis, FMT was performed after 42 days, and all participants experienced rapid resolution of symptoms (42). These cases suggest that FMT could effectively reduce inflammation and immunosuppression during sepsis, particularly when the infections are associated with intestinal issues. However, a major limitation is that to use FMT widely, antibiotic treatment should be discontinued (44). It is challenging to reach a consensus on antibiotic discontinuation in critically ill patients, as antibiotics are essential for treatment (45).

The safety of FMT has not been confirmed in immunocompromised recipients, and most FMT trials have excluded these high-risk participants. A recent review of 44 studies on FMT for CDI found that 88% of immunocompromised patients (mostly on immunosuppressive medication) achieved successful treatment (46). This success rate suggests that FMT is equally safe in immunocompromised patients as in those with a healthy immune system (46). However, the risk of transferring live microorganisms to recipients with underlying illnesses remains higher (47, 48).

According to most guidelines, pregnancy is considered a contraindication for FMT (32). Despite a lack of evidence on implementing FMT during pregnancy, Saeedi et al. reported a case of successful use of FMT in a pregnant patient with CDI (49). However, clinical studies are required regarding the safety and efficacy of FMT in pregnant patients.

## 7 Adverse effects

FMT is generally safe and effective for treating various conditions, but it’s important to weigh the risks and benefits for each patient and monitor for adverse effects during and after the procedure (50). The adverse effects of FMT can vary depending on factors such as the donor’s health, the recipient’s immune system, and the administration route. Adverse reactions following FMT can range from mild to severe. Adverse events are mostly mild and involve GI symptoms, but serious complications such as perforation, bacteremia, sepsis, multi-organ failure, and death have also been reported (50–52).

Based on a systematic review and meta-analysis, serious adverse events of FMT occur in less than 1% of patients and include colectomies, bacteremia/infections, hospitalizations, life-threatening complications, and deaths (53). In another review, the total incidence rate of adverse events of FMT was 28.5%, with abdominal discomfort being the most common (54). FMT-related adverse effects are usually short-term and gastrointestinal. For example, a study reported transmission of *Shiga toxin-producing Escherichia coli* following FMT (51). Knowledge of these events’ prevalence and clinical presentation is necessary for their timely diagnosis (**Table 1**).

**Table 1:** Relative and absolute contraindications of FMT.

Relative Contraindications	Absolute Contraindications
‐ Recent Gastrointestinal Surgery ‐ Severe Acute Illness ‐ Pregnancy and Lactation ‐ Pediatric patients ‐ Elderly patients	‐ Severe Immunocompromise ‐ Gastrointestinal Obstruction ‐ Toxic megacolon ‐ Recent major surgery ‐ GI tract perforation

## 8 Antibiotic-associated dysbiosis

Antibiotics can significantly impact the gut microbiota, leading to the expansion of harmful bacteria and causing dysbiosis, which is associated with various diseases, including antibiotic-associated dysbiosis (AAD) (55). Studies suggest that dysbiosis induced by antibiotic exposure can lead to conditions such as CDI and IBD (56, 57). Studies have also explored the role of FMT in combating multi-drug resistant pathogens, such as CDI, or in the case of ADD affecting lung infections (58, 59). FMT has been shown as a potential intervention to restore microbial balance and alleviate symptoms in the context of AAD, where antibiotic treatment disrupts the gut microbiota. While antibiotics are commonly used to treat AAD, they can have limited efficacy and may lead to dysbiosis (60).

## 9 *Clostridioides difficile* Infection

CDI is the most prevalent healthcare-associated infection, with an alarming increase in the occurrence, recurrence, severity, and mortality rates in recent years (61). *Clostridioides difficile* is a gram-positive bacterium that forms spores and is a leading cause of nosocomial infections. These infections, triggered by an imbalance in the gut microbiota due to antibiotic use, can lead to various symptoms, including diarrhea, dehydration, colitis, and toxic megacolon (60, 62, 63).

Despite the widespread nature of CDI, significant progress has been made in developing new therapies and prevention methods based on updated practice guidelines. FMT is a highly effective alternative to antibiotics for treating recurrent CDI (59). Despite various challenges, such as time-consuming procedures and difficult administration routes, FMT has shown success in treating CDI with minimal side effects, even in immunocompromised patients (64, 65).

Studies suggest FMT as an effective treatment for CDI patients, as well as recurrent or refractory infections, even in immunocompromised patients who are highly susceptible to C. difficile contamination (44, 60, 66). A retrospective cohort study investigated FMT’s efficacy in treating recurrent CDI and reported this method as a promising approach with a success rate of 60% (67). Also, in 2018, Shogbesan et al. reviewed articles on FMT administration to treat CDI in immunocompromised patients, including individuals who took immunosuppressant drugs, underwent chemotherapy, had human immunodeficiency virus, immunodeficiency disorders, or underwent organ transplantation (46). These findings support using FMT as a treatment for CDI in immunocompromised patients. They also reported that the rates of serious adverse events in immunocompromised patients were comparable to those in immunocompetent patients. However, due to the diverse range of immunosuppression subtypes, the authors could not draw definite conclusions regarding the response to FMT in any immunocompromised condition or combination. Furthermore, a multicenter cohort study examined the effect of FMT on Clostridium relapse in immunocompromised pediatric patients. They observed a substantial success rate comparable to that of immunocompromised adults and immunocompetent children (68).

While FMT is highly effective, it has considerable drawbacks, including the risk of infections and the lack of extensive long-term safety data (69, 70). Researchers have emphasized the necessity of monitoring and following up with patients undergoing FMT for the possible observed side effects. Additionally, repeating FMT, if needed, can lead to complete recovery, fewer relapses, and reduced side effects (63, 66). It is worth noting that, like any medical procedure, FMT has its limitations, but when used as indicated, it provides a safe alternative for treating CDI (**Table 2**).

**Table 2:** Effects of FMT on CDI.

Author/year	Study Design	CDI severity	FMT administration	Result	Conclusion
Kelly et al. 2014 (71)	Case-series	SFCDI	Lower endoscopy	*Discharging after 3.5 weeks *CDI cure rate after a single FMT: 78% *Overall cure rate: 89% *Disease flare post FMT:14% *SAE within 12 weeks post FMT: 15%	This series demonstrates the effective use of FMT for treating CDI in high-risk IC patients with minimal SAEs or related AEs. Importantly, no infectious complications were observed in these patients.
Zaniah et al. 2015 (72)	Retrospective	SFCDI	Upper GI + lower endoscopy + enema	* Cure after IMT: 79% * Recurrence: 0 * Die as a result of CDI or IMT: 0	effective and safe for treating severe and refractory CDI, and prevents recurrence.
Aroniadis et al. 2016 (73)	Case-series	SFCDI	Lower endoscopy	* Primary cure rate: 88.2% * Late CDI recurrence (≥90 d): 5.9% * Adverse effects directly related to FMT: 0 * Diarrhea resolve: 75% * Diarrhea improve: 25% * Abd pain resolve: 72.7% * Abd pain improve: 27.3%	In this group of patients with severe or complicated CDI, FMT demonstrated both effectiveness and safety. The primary cure rate was 88.2%, while the secondary cure rate reached 94.1%.
Gundacker et al. 2017 (74)	Case-Series Retrospective (chart review)	F	Upper GI + lower endoscopy + enema	* Eventual cure:6p * Death attributed to CDI: 1p	FMT is a viable alternative that should be considered for patients who are not eligible for surgery. In cases where lower endoscopy is not feasible, administering FMT through a nasogastric tube should also be considered an option.
Allegretti et al. 2018 (75)	Prospective Cohort	SFCDI	Lower endoscopy	* FMT failure: 16.7% * The mean failure time: 14.8 ± 12.5 d	The proposal suggests contacting patients one week after FMT to assess primary nonresponse and provide symptom guidance. Additionally, a follow-up call or clinic visit at week 4 is recommended to identify early secondary nonresponse. This protocol could expedite treatment by detecting FMT failures sooner.
Hocquart et al. 2018 (76)	RCT	SFCDI	Lower endoscopy	* FMT improved survival in severe cases (OR, 0.08 [95% CI,.016–.34], P = .001) but not in nonsevere cases (OR, 1.07 [95% CI,.02–56.3], P = .97), independent of age, sex, comorbidities (Charlson score), and ribotype	Proposing early FMT as a primary treatment for severe CDI can significantly reduce mortality rates.
Alukal et al. 2019 (77)	Clinical Trial	S	Upper GI + lower endoscopy	* The primary cure rate after a single round of FMT: 78% * Avoided a colectomy during the same hospital admission: 88.88% * CDI-related death rate: 12.5%	The positive outcomes observed in our use of FMT for fulminant CDI indicate that this treatment method holds significant promise as an alternative to colectomy. Moreover, it can potentially be a beneficial intervention for preserving bowel function.
Nowak et al. 2019 (67)	Retrospective Cohort		Rectal administration: (42/47) p + through a nasogastric tube: (5/47) p	*Cured after one treatment: 53% *Cured after 2–4 FMTs: (7/47) *Overall cure rate: 68% *Cure rates of male: 86% *Cure rates of female: 60% *SADE: 0	Data suggests that frozen fecal cultures can be a viable and effective alternative to fresh donor feces in treating recurrent CDI.
Cheng et al. 2019 (78)	Retrospective	SFCDI	Enema vs. Colonoscopy	* CDI-related mortality after FMT: 4.4% * Among 430 hospital admissions with severe or fulminant CDI, 205 admissions occurred before (pre-FMT) and 225 after FMT program implementation (post-FMT)	In hospitalized patients with SFCDI, the implementation of an FMT program resulted in a significant reduction in CDI-related mortality.
Tixier et al. 2020 (79)	Prospective Cohort	SFCDI	Upper GI + lower endo capsulescopy + enema	* Decrease in odds for mortality: 77% (OR 0.23, 95% CI 0.06-0.97)	In critically ill patients with severe and fulminant C. difficile infection, fecal microbiota transplantation (FMT) offers a significant advantage in reducing mortality compared to the standard of care. Therefore, FMT should be considered a treatment option for these patients.
Conover et al. 2023 (68)	Retrospective Cohort	NR	Colonoscopy + Nasogastric tube + Nasointestinal tubes + Capsule	* Success rate after first FMT: 79% *Success rate after one or more FMT: 86% *SAEs: 31% *Deaths or infections with multidrug-resistant organisms: 0	FMT has a high success rate for recurrent CDI in pediatric IC patients, similar to adults and immunocompetent children. However, it is crucial to carefully evaluate the risks and benefits due to the occurrence of FMT-related serious adverse events (SAEs) at a rate of 9.5%.
Parcari et al. 2023 (65)	Retrospective Cohort	NR	Colonoscopy	*Overall FMT cured rCDI: 91% *Remission or an amelioration of UC activity: 69%	study shows that FMT is highly effective in curing rCDI without severe adverse events. Repeat FMT is associated with CDI cure and leads to remission or improvement of UC activity. These findings support the routine use of sequential FMT in UC patients with rCDI.

NR, non-reported; CDI, clostridium difficile infection; rCDI, recurrent clostridium difficile infection; SFCDI, Severe Focal *Clostridioides difficile* Infection.

## 10 Safety and efficacy of FMT on GI diseases and disorders

### 10.1 Inflammatory bowel disease

IBD refers to chronic inflammatory conditions affecting the GI tract, mainly including ulcerative colitis and Crohn’s disease (80). FMT shows promise as a therapeutic option for inducing remission in IBD, particularly when repeated dosing and antibiotic pre-treatment strategies are employed. Its efficacy is linked to modulation of the gut microbiome composition and restoration of microbial diversity. While generally safe, the risk of IBD flare after FMT needs to be considered (81, 82).

#### 10.1.1 Ulcerative colitis

Ulcerative colitis is a type of IBD affecting the colon and rectum. Dysregulated gut microbiota can compromise the immunomodulatory function of the gastrointestinal system and contribute to the progression of Ulcerative Colitis. Therefore, FMT could be a helpful alternative therapeutic option to control the microbiota’s imbalance (83). Results from both human and animal studies highlight FMT’s efficacy in patients with ulcerative colitis (84–86).

FMT leads to higher rates of clinical remission and endoscopic improvement in patients with active ulcerative colitis compared to standard therapy alone (86). In one study, 23.8% of patients who underwent a second course of FMT achieved a longer clinical response compared to those with poor adherence (87). A recent systematic review and meta-analysis study conducted by Chehade N et al. concluded that FMT is an effective therapeutic option for inducing clinical remission, clinical response, and endoscopic remission in patients with active ulcerative colitis, mainly when multiple FMT administrations are employed. These findings support the potential role of FMT as a treatment modality for ulcerative colitis (85).

#### 10.1.2 Crohn’s disease

Crohn’s disease, another main category of IBD disorders, can affect any part of the gastrointestinal tract (from the stomach to the anus) (88). Similar to ulcerative colitis, gastrointestinal microbial dysregulation plays a prominent role in the occurrence and development of this disease (89). Systemic immunosuppressive modalities are considered the preferred treatment option for patients with Crohn’s disease; however, less than half of those undergoing standard treatment achieve remission, resulting in high morbidity and mortality rates (90, 91).

FMT might be an alternative treatment option for these patients, with beneficial outcomes. Several studies evaluated the effect of FMT on clinical and endoscopic remission of Crohn’s disease patients. A recent RCT revealed a higher clinical remission rate in the FMT group compared with the control group at both 10 and 20 weeks of follow- Crohn’s disease (87.5% vs. 44.4% at week 10 and 62.5% vs. 33.3% at week 20). Also, patients with Crohn’s disease who underwent FMT showed significant improvement in endoscopic remission compared to the control group (92). Another RCT investigated the differences in clinical remission between individuals who received FMT by gastroscopy and those who received it by colonoscopy. The authors declared no significant difference between the two groups. Similarly, no substantial improvement regarding endoscopic remission was reported in either of the groups (93).

Additionally, FMT is generally a safe option in patients with Crohn’s disease with no documented serious side effects. However, more extensive clinical trials are required to establish the safety and efficacy of this therapeutic intervention among patients with Crohn’s disease (89).

### 10.2 Inflammatory bowel syndrome

Inflammatory bowel syndrome (IBS) is a functional GI disorder that significantly impacts a person’s quality of life. The exact pathogenesis of IBS is unknown (94); however, recent studies have highlighted the effect of GI microbial imbalance on this disease (95–98). Several studies investigated the impact of FMT in patients with IBS and compared the outcomes with those of a control group over various follow-up periods. Results showed that the IBS severity scoring system decreased prominently within three months of using FMT (by almost 50-75 points or more) (99–104). A randomized controlled study evaluated symptom improvement over three months, reporting that 64% of the patients who received FMT experienced improvement, compared to 42% in the control group. However, this difference was not statistically significant (105). IBS patients treated with FMT were compared to those in the control group in terms of adverse reactions. Results showed no statistically significant difference between the groups (35% vs 26%, P = 0.62), and most of the reported adverse events were mild, transient, and related to the gastrointestinal system (106).

## 11 Safety and efficacy of FMT on obesity and metabolic syndrome

FMT has recently gained popularity for treating and preventing various infections, especially gastrointestinal ones like CDI (9, 107). However, its potential goes beyond infections, as it’s now being explored for non-infectious conditions like metabolic diseases. With the global rise in diabetes, metabolic syndrome, and obesity, treatment options, including FMT, are evolving as well (108–110). Animal studies have shown that FMT can be effective in preventing obesity and metabolic diseases by mechanisms like increasing fat breakdown and altering gut bacteria levels (111, 112).

Recent studies suggest that FMT could effectively treat obesity and metabolic syndrome by improving glycemic control, insulin sensitivity, and lipid profile in the short term. The recipient’s initial gut microbiome and the engraftment of donor microbiota may impact the metabolic response to FMT (113, 114).

In a study by Qiu B et al. (113), the role of gut microbiota dysbiosis in the pathogenesis of obesity was investigated. This study concluded that intestinal dysbiosis contributed to metabolic dysregulation in obese individuals. Significantly, transplantation of healthy intestinal flora through FMT successfully reversed dysbiosis and improved gut barrier function and metabolic inflammation, ultimately ameliorating abdominal fat deposition (113). In a systematic review of 334 patients with obesity and metabolic syndrome, FMT was shown to positively affect several metabolic indicators. After undergoing FMT, patients experienced improvements in caloric intake, fasting glucose levels, HOMA-IR (a measure of insulin resistance), blood pressure, total cholesterol, and inflammatory markers. However, despite these benefits, some obesity-related parameters increased post-FMT (115). Another comprehensive meta-analysis included 9 studies with 303 participants. Short-term outcomes (<6 weeks after FMT) indicated that FMT was associated with lower fasting blood glucose, HbA1c, and insulin levels, along with higher levels of high-density lipoprotein cholesterol compared to the placebo group (113).

## 12 Safety and efficacy of FMT on diabetes mellitus

FMT shows promise as a potential treatment for type 2 diabetes mellitus (T2DM) by improving insulin resistance and blood glucose control and modulating the gut microbiome. While generally safe, close monitoring is recommended, especially in older patients and those with inflammatory bowel disease, as they may be at increased risk for side effects after FMT (116, 117).

Wu et al.’s study (116) We have investigated the effects of FMT on reversing insulin resistance in patients with T2DM. This randomized controlled study demonstrated that FMT alone or combined with metformin effectively reversed insulin resistance, improved glycemic control, and modulated the gut microbiome composition in patients with newly diagnosed T2DM. The study highlights the potential therapeutic role of FMT in managing T2DM by targeting insulin resistance and gut dysbiosis (116).

Almost all studies on FMT admit its safety and tolerability among this population (118). Some studies have not shown significant differences in the results of the FMT groups, including pre-operative and post-operative weight, insulin and glucose levels, and insulin sensitivity (119–123). It is shown that FMT is only effective in certain patients with T2DM who have specific levels of bacterial markers in their microbiota, such as *Anaerotruncus Ruminococcaceae* and *Rikenellaceae* family (124). Therefore, the controversies may be due to individual differences, such as variable intestinal microbiota or laboratory settings. Future research should aim to unify the mentioned cases and conditions and re-examine the results from a more comprehensive point of view.

## 13 Safety and efficacy of FMT in allergic diseases

Allergic reactions are our body’s response to specific allergens by releasing antibodies (125). Gut microbiota plays a crucial role in our immune response to allergens. Therefore, any intestinal microbial dysregulation might affect the development of allergic disorders such as celiac, asthma, and other allergies (126, 127).

### 13.1 Food allergy

Food allergy, one of the most prevalent types of allergic disorders, occurs in response to specific food allergens (128). The primary treatment strategy for such disorders is avoiding specific foods that may cause allergic reactions, leading to limited dietary diversity and impaired quality of life (129). Exploring new treatment strategies, such as FMT, can help improve symptoms in this particular group (130). Based on a systematic review by Jensen et al. (131), FMT led to increased tolerance to allergenic foods in some studies involving patients with peanut, cow’s milk, and multiple food allergies. FMT resulted in changes in the gut microbiome composition of FA patients, with increased diversity and abundance of potentially beneficial bacterial taxa. Factors like donor selection criteria, FMT preparation method, recipient characteristics, antibiotic pre-treatment, and repeated FMT dosing were associated with better outcomes in some studies. No serious adverse events related to FMT were reported in the included studies (131).

### 13.2 Allergic rhinitis

Recent investigations have highlighted FMT as a potential therapeutic avenue for allergic diseases, including allergic rhinitis. Studies addressing the relationship between gut microbiota diversity and allergic sensitization can help us understand the underlying mechanisms of FMT in managing allergic rhinitis (132–134). It is shown that reduced gut microbial diversity and alterations in specific bacterial taxa may contribute to the development of allergic rhinitis by modulating immune responses and promoting allergic inflammation. Individuals with allergic rhinitis had lower gut microbial diversity and distinct gut microbial compositions compared to healthy controls, which was associated with an increased risk of developing allergic rhinitis and sensitization to major inhaled allergens (132–134).

Dong et al. (135) studied the effects of FMT on allergic rhinitis and its potential mechanisms, showing that FMT can alleviate symptoms of allergic rhinitis in a mouse model by restoring gut microbiota diversity and composition, which modulates the balance between Th2 cells and regulatory T cells, ultimately suppressing allergic inflammation (135). Zou et al. (136) studied the long-term safety and effectiveness of FMT in 74 children. Initial remission rates were reasonable but declined over time. Some patients developed new conditions like rhinitis and constipation. Short-term adverse events occurred in 13.7% of patients, primarily within two days post-FMT. Long-term follow-up (up to 7 years) showed no development of autoimmune, metabolic, or rheumatologic disorders, or tumors. The primary clinical remission rate after FMT was 72.9% but gradually decreased over time. Nine children developed rhinitis, five developed rhinitis and were underweight, and six developed constipation during the follow-up period (136).

### 13.3 Asthma

Asthma, a rapidly increasing allergic disorder, is predicted to affect about 100 million more individuals by 2025 (137). The role of the microbiome in asthma pathogenesis and treatment responsiveness has received significant attention (138). The link between dysbiosis, immune dysregulation, and disease exacerbation implicates gut microbiota alterations in asthma pathogenesis. Studies show a complex association between gut microbiota composition and asthma outcomes, with specific taxa like *Lachnospiraceae* and *Oscillospiraceae* serving as potential biomarkers of disease severity and progression (139). Excess fungi such as Candida are also linked to asthma susceptibility and exacerbation (140). While prebiotics and dietary interventions show promise in modulating microbiota and reducing asthma risk, further investigation is needed to confirm their efficacy (138).

An animal study by C Wu et al. (141), demonstrates that FMT can alleviate ovalbumin-induced allergic airway inflammation in a neonatal mouse model of asthma, potentially through modulation of the gut microbiota, enhancement of regulatory T cell responses, and regulation of the PD-1/PD-L1 axis, which is involved in immune tolerance and suppression of allergic inflammation (141). In their review article, Kang and Cai (142) showed that gut microbiome dysbiosis, characterized by reduced diversity and altered composition, is associated with the development and exacerbation of asthma (142).

### 13.4 Dermatitis

Several studies on FMT have shown promising results as a potential treatment for atopic dermatitis, a chronic inflammatory skin disease (143–145). The pathogenesis of atopic dermatitis involves complex factors, including gut microbiota and immune modulation, which remain poorly understood. The gut microbiome plays a vital role in modulating immunity and skin health, and dysbiosis (imbalance) in the gut microbiota is associated with the development of atopic dermatitis (146). A study aimed to restore gut microbiota via FMT to ameliorate atopic dermatitis in mice. FMT resulted in the restoration of gut microbiota to the donor state, increases in the levels of gut metabolites, restoration of the Th1/Th2 balance, and reduction of atopic dermatitis-induced allergic responses. FMT shows potential as a new therapy for atopic dermatitis (145). In a murine model of atopic dermatitis induced by calcipotriol exposure, FMT showed a notable trend toward reversing the epidermal layer thickening, suppressing inflammatory cytokines, and mitigating atopic dermatitis-related inflammation (144). A pilot study evaluated the efficacy of a single oral FMT capsule in dogs with atopic dermatitis. The results showed a significant reduction in atopic dermatitis severity scores and pruritus after FMT treatment, indicating its potential as a novel therapy for canine atopic dermatitis (147). These studies highlight the connection between gut microbiota and dermatitis, suggesting the therapeutic use of FMT and probiotics to mitigate symptoms. Further human studies are needed to understand the mechanisms and optimize dermatitis treatment.

## 14 Autoimmune rheumatic diseases

### 14.1 Rheumatoid arthritis

RA is an autoimmune disease that causes synovial tissue inflammation and joint symptoms. Genetic and environmental factors influence the development of RA. Despite known contributing factors, the exact cause of RA is still unclear (148). Evidence suggests that mucosal immunity, influenced by the interactions between gut microbiota and host, plays a crucial role in RA development. RA starts in mucosal sites and then involves synovial joints through the gut-joint axis (149). Despite an emphasis on the gut microbiota variations between RA patients and control groups, current data on the diversity and richness of species in clinical research is inconsistent and variable (150).

Several clinical studies on RA patients have reported changes in microbial diversity (151, 152). For instance, research suggests that *Porphyromonas gingivalis* may contribute to arthritis by inducing the production of anti-citrullinated protein antibodies and causing inflammation (153). In a study of 126 participants, RA patients showed higher *Bacteroidetes* and lower *Actinobacteria*, *Firmicutes*, and *Proteobacteria* compared to healthy individuals (154). In contrast, a rise in *Actinobacteria* was documented in a separate investigation (155). Consequently, FMT can be a helpful intervention in RA treatment by correcting imbalances in the microbiota.

The effectiveness of FMT in RA treatment is debated. In a murine model, germ-free mice received FMT from RA or IBD donors, resulting in physical changes like altered cartilage, paw deformities, increased inflammatory mediators, and activated T-lymphocytes. Behavioral modifications, occult bleeding, and gut disruption were also observed, highlighting the interconnectedness of gut microbiota, the immune system, and the gut-brain axis (156). Limited human studies exist regarding the safety, efficacy, and tolerability of FMT in RA patients. A case study reported positive outcomes in a 20-year-old woman with RA following FMT treatment from a healthy 8-year-old donor. The patient showed improvements in disease activity, disability index, and rheumatoid factor titer without discomfort (157). More research through prospective RCTs is needed to explore the potential of FMT for treating RA.

### 14.2 Systemic sclerosis

Systemic sclerosis is an autoimmune condition that causes fibrotic alterations in internal organs and skin and vascular anomalies (158). Most patients experience GI difficulties, which cause symptoms such as dysphagia, reflux, stomach discomfort, malnutrition, incontinence, and diarrhea, affecting their quality of life and mental health (159). Research shows that gut microbiota disruption is observed in individuals with Systemic sclerosis. Numerous cohort studies have demonstrated notable variations in gut microbiota between individuals with Systemic sclerosis and those in good health (159, 160). An observational cohort study in Sweden identified distinct microbiota variations in fecal specimens obtained from 98 individuals diagnosed with Systemic sclerosis (161). Additionally, a study on the microbiota variations among patients with Systemic sclerosis indicated a decrease in specific beneficial commensal genera like *Bacteroides, Faecalibacterium*, and *Clostridium*, while potentially pathogenic genera, such as *Fusarium* and *Ruminococcus*, were observed to increase (162). However, more comprehensive RCTs are essential to examining the therapeutic effect and safety of FMT in Systemic sclerosis.

### 14.3 Systemic lupus erythematosus

SLE is a chronic autoimmune illness in which the immune system targets cell nuclei, resulting in the development of autoantibodies that assault organs (163). SLE pathogenesis is hypothesized to be impacted by genetics, hormones, environment, and other factors (164). Several human and animal investigations have shown that the gut microbiota composition of SLE patients is changed, with lower *Lactobacillaceae* and higher *Lachnospiraceae* (165, 166). Furthermore, probiotics such as bifidobacteria and lactobacilli are reduced, whereas E. coli levels rise in SLE patients (164). Notably, a pilot clinical trial in 20 SLE patients demonstrated that FMT, in the form of oral capsules, improved clinical parameters of SLE by restoring gut microbiota, increasing SCFAs, and reducing IL-6 levels and CD4+ memory/naïve T cell ratio. These positive changes in gut microbiota were sustained for up to 12 weeks with no severe side effects (167).

### 14.4 Sjogren’s syndrome

Sjogren’s syndrome involves inflammation and an autoimmune response, leading to dry eyes and mouth due to gland dysfunction. Genetic and epigenetic factors can affect its onset (168, 169). Studies on mice tested the application of FMT. Another study found that FMT in mice improved dry eye symptoms by reducing corneal damage and increasing goblet cell density (170).. In a recent clinical trial, individuals with immune-mediated dry eye symptoms received donor FMT by enema. 80% of the participants experienced shifts in their gut microbiota composition towards that of the donors. Although there was a subjective improvement in dry eye symptoms for half of the patients, the recipients’ microbiota remained somewhat different from the donors’, with no significant changes noted (171). The impact of bacterial taxa on sjogren’s syndrome is unclear. FMT has shown some effectiveness in alleviating symptoms among patients with sjogren’s syndrome; more human studies are needed in this field.

### 14.5 Psoriatic arthritis and psoriasis

Psoriatic arthritis is a chronic and progressive immune-mediated disorder with various clinical features that typically affects adults with a history of psoriasis (172). Psoriasis could be linked to genetic, immune, and environmental factors (173). Gut microbiota strongly impacts the immune system and may affect autoimmune diseases like psoriatic arthritis. Recent research has explored using FMT to treat psoriatic arthritis (174). Research has shown that psoriasis development is linked to the T helper cell (Th)17/IL-23 axis, and gut microbiota composition may influence T cell maturation. Segmented filamentous bacteria can trigger inflammatory reactions in Th17 cells in the GI tract (175). Additionally, microbiota can produce SCFAs, which have regulatory effects on T cells in an inflammatory environment driven by T cell activity (176, 177). A study confirmed the safety of FMT application via the duodenal route in 10 patients with psoriatic arthritis. Some experienced mild adverse effects, but no life-threatening effects were observed (178). Another report showed symptom improvement in a 36-year-old patient with severe plaque psoriasis and IBS after receiving two episodes of FMT without adverse reactions (179). However, in another study on patients with active peripheral psoriatic arthritis, the FMT group had a higher treatment failure rate than the placebo group, and the overall success rate was more significant in the placebo group. No severe adverse effects were reported in either group (180). These studies suggest a strong link between gut microbiota and the immune effects of psoriatic arthritis. FMT holds promise as a potential treatment for psoriatic arthritis patients, but further investigation is needed to confirm its efficacy in this population.

## 15 Safety and efficacy of FMT in organ transplant

Organ transplantation has progressed from an experimental 20th-century strategy to a proven solution for end-organ failure (181). In the first-year post-transplant, recipients face significant issues, particularly multidrug-resistant infections. Preventative measures and careful monitoring are essential to improve outcomes for these high-risk patients (182). Research suggests that FMT may help restore gut microbial balance and minimize problems in patients receiving stem cell or organ transplants (183). Studies have indicated that FMT is both safe and effective in treating recurrent CDI in transplant patients, with few significant side effects observed (184–187). A study was conducted on two cases with lung and renal transplantation, with recurrent CDI post-transplantation. Both patients underwent two FMTs, resulting in complete symptom resolution without infectious complications (184).

Research indicates that FMT could help re-establish the equilibrium of the gut microbiota and avert difficulties in patients receiving allogeneic hematopoietic stem-cell transplantation for cancers of the blood system (183). Numerous investigations into the efficacy and safety of FMT in patients receiving organ transplants have reported encouraging findings. Several studies have shown that FMT is safe and effective in treating recurrent CDI in organ transplant recipients (184–187). Due to the compromised immune system in organ transplant recipients compared to the general population, FMT faces more safety concerns, but serious adverse events are still uncommon (184–186).. organ transplant patients may face challenges like infections, viral reactivation, and the need for careful monitoring of immunosuppressive therapy (181). Adverse events like bacteremia, cytomegalovirus reactivation, and allograft rejection are rare. Reported adverse events include self-limiting conditions like nausea, abdominal pain, and FMT-related diarrhea (186). There is a potential increased risk of procedure-related serious adverse events (185). Two studies found that FMT may require additional antibiotics or repeat procedures to maximize cure rates in organ transplant patients. The overall cure rate after subsequent FMT was 91.3% (186). In pediatric organ transplant recipients, 83.3% of single FMT were successful, but some patients required multiple FMT, and one experienced serious adverse effect (185). In conclusion, studies indicate that FMT can usually be safe and effective in organ transplant recipients; however, since this population is immunocompromised, frequent monitoring for potential problems is necessary.

## 16 Conclusions, clinical challenges, limitations, and prospects of FMT

FMT is gaining recognition as a promising treatment for conditions tied to gut dysbiosis, such as CDI, and potentially other issues like diabetes, obesity, and autoimmune disorders. Many studies have shown that FMT can be safe and effective across various diseases, offering hope to patients who may have exhausted other options.

However, bringing FMT into mainstream clinical practice has its challenges. There’s a pressing need for standardized procedures, careful donor screening to reduce risks, and strategies to handle the rare but serious complications that can arise. While the potential of FMT is undeniable, fully understanding how it works and refining its use in the clinic is still a work in progress.

The future of FMT looks bright, with impressive success rates and growing support among healthcare providers. Yet, the varying results in patients underscore the importance of developing consistent treatment approaches and gaining a deeper understanding of what makes FMT effective. These challenges are also opportunities for further research, including larger clinical trials, advanced studies of fecal metabolites, and animal model testing, all of which could shed light on the complex ways FMT can help heal. Continued research will ensure long-term safety and perfect delivery methods and confirm its effectiveness for many patients.
